# Canine Gastric Carcinomas: A Histopathological and Immunohistochemical Study and Similarities with the Human Counterpart

**DOI:** 10.3390/ani11051409

**Published:** 2021-05-14

**Authors:** Alexandros Hardas, Alejandro Suárez-Bonnet, Sam Beck, William E. Becker, Gustavo A. Ramírez, Simon L. Priestnall

**Affiliations:** 1 Department of Pathobiology & Population Sciences, The Royal Veterinary College, North Mymms, Hatfield, Hertfordshire AL9 7TA, UK; asuarezbonnet@rvc.ac.uk (A.S.-B.); wbecker6@rvc.ac.uk (W.E.B.); spriestnall@rvc.ac.uk (S.L.P.); 2 VPG Histology, Horfield, Bristol BS7 0BJ, UK; sam.beck@synlab.co.uk; 3 Department of Animal Science, School of Agriculture, Food Science and Veterinary Medicine (ETSEA), University of Lleida, 25198 Lleida, Spain; gustavo.ramirez@udl.cat

**Keywords:** canine, stomach, gastric carcinoma, *Helicobacter* spp., p16, 14-3-3σ, E-cadherin and CD44

## Abstract

**Simple Summary:**

Gastric carcinoma (GC) continues to be one of the leading causes of death in humans and is the most common neoplasm in the stomachs of dogs. In both species, previous studies have demonstrated that the disease is heterogeneous, with genetic and environmental factors playing a quintessential role in disease pathogenesis. Compared to humans, the incidence of gastric carcinoma in dogs is low although, in a small number of breeds, a higher incidence has been reported. In dogs, the etiology and molecular pathways involved remain largely unknown. This retrospective study reviews current signalment data, evaluates the inflammatory component and association with *Helicobacter* spp. presence in various canine gastric carcinoma histological subtypes, and investigates potential molecular pathways involved in one of the largest study cohorts to date. The benefit of such a comparative study is to highlight the parallel histological features and molecular pathways between dogs and humans.

**Abstract:**

Canine gastric carcinoma (CGC) affects both sexes in relatively equal proportions, with a mean age of nine years, and the highest frequency in Staffordshire bull terriers. The most common histological subtype in 149 CGC cases was the undifferentiated carcinoma. CGCs were associated with increased chronic inflammation parameters and a greater chronic inflammatory score when *Helicobacter* spp. were present. Understanding the molecular pathways of gastric carcinoma is challenging. All markers showed variable expression for each subtype. Expression of the cell cycle regulator 14-3-3σ was positive in undifferentiated, tubular and papillary carcinomas. This demonstrates that 14-3-3σ could serve as an immunohistochemical marker in routine diagnosis and that mucinous, papillary and signet-ring cell (SRC) carcinomas follow a 14-3-3σ independent pathway. p16, another cell cycle regulator, showed increased expression in mucinous and SRC carcinomas. Expression of the adhesion molecules E-cadherin and CD44 appear context-dependent, with switching within tumor emboli potentially playing an important role in tumor cell survival, during invasion and metastasis. Within neoplastic emboli, acinar structures lacked expression of all markers, suggesting an independent molecular pathway that requires further investigation. These findings demonstrate similarities and differences between dogs and humans, albeit further clinicopathological data and molecular analysis are required.

## 1. Introduction

Gastric carcinoma is a rare form of cancer in domestic animals, and in dogs accounts for <1% of all reported neoplasms [1], with adenocarcinoma the most frequent (50–90%) [1]. The median age of gastric carcinoma development is 10 years, with rough collies, Staffordshire bull terriers, chow-chows, Belgian shepherds, Norwegian Lundehunds, Cairn terriers and West Highland white terriers being the breeds most likely to be affected [2].

Compared to gastric carcinoma in humans, the incidence of canine gastric carcinoma (CGC) is relatively low; however, in recent years the disease has been more frequently diagnosed [3,4]. Considering the proposed causal effect of diet on gastric neoplasia in humans [5], this increased frequency in dogs over the last 30 years could be similarly attributed. Breed predisposition has also contributed [6], with those breeds at increased risk becoming more popular. In addition, increased longevity and advances in veterinary diagnostic techniques, such as gastroscopy, may have also contributed to increased diagnosis.

Clinical signs of CGC include vomiting that may progress to hematemesis, melaena, anemia, lethargy, ptyalism, polydipsia, abdominal distension, and abdominal discomfort. Prognosis is generally poor, with a median survival time of 35 days, and confirmed metastasis in about 70–90% of cases at the time of diagnosis or death [7]. Common sites of metastasis include the gastric lymph nodes, omentum, liver, duodenum, pancreas, spleen, esophagus, adrenal glands and lungs [2].

Classification of gastric carcinomas in dogs follows the World Health Organization (WHO) [8] scheme, adapted from humans, which is based on the predominant histological features and the main patterns of cells within the neoplasm: papillary, tubular, mucinous, signet-ring cell (SRC) and undifferentiated types [2]. An alternative scheme, again adapted from human medicine, the Lauren classification, divides tumors into intestinal—cohesive masses and tubular structures; diffuse—individual or scattered nests of neoplastic cells; and mixed—incorporating features of both intestinal and diffuse types [9]. Both schemes have been applied in previous studies [1,3].

The association between chronic inflammation, caused by a variety of factors (bacterial, viral, and parasitic infections, chemical irritants, and nondigestible particles), and carcinogenesis is now well established in humans and animals [10]. The risk of carcinogenesis is higher, the longer the inflammation persists [11]. In humans, it has been shown that several risk factors, such as *Helicobacter pylori* infection, diet, and smoking, are involved in the precancerous cascade of events that lead to gastric adenocarcinomas [12].

The pathogenesis of CGC remains elusive, albeit a high prevalence in certain breeds (e.g., Staffordshire bull terrier, Norwegian Lundehund and Belgian shepherd dog) suggests an underlying genetic etiology [6]. A clear role for *Helicobacter* spp., similar to *H. pylori* in humans, has not been reported in domestic species to date [13]. Whether other *Helicobacter* spp. are involved with carcinogenesis is unclear. *H. pylori* has occasionally been recognized in the canine stomach [2,12], however, the predominant species in dogs are *H. felis*, *H. bizzozeronii* and *H. heilmannii* [14,15]. A clear association between gastric inflammation and *Helicobacter* spp. presence has not been made in previous studies, and in addition, an association with gastric carcinoma has also not been investigated [16].

The role of cell cycle regulators and cell adhesion molecules in cancer is complex and paradoxical, varying by cell type and stage of tumorigenesis [17]. In this study, we aimed to examine the involvement of important cell cycle regulators and cell adhesion molecules, previously studied in human gastric carcinoma, in dogs.

E-cadherin is a calcium-dependent cell–cell adhesion molecule that preserves epithelial integrity and can act both as a tumor-suppressor and as an oncoprotein [18,19]. A recent large-scale study separating subtypes according to their growth pattern (polypoid or non-polypoid, i.e., signet cell, mucinous and undifferentiated carcinoma) showed that there is a complete loss of E-cadherin in non-polypoid and undifferentiated carcinomas, and reduced expression in polypoid, with no evidence of malignant alteration or invasion in canine gastrointestinal tumors [20].

CD44 is a cell surface receptor for hyaluronic acid and binds to collagen, fibronectin and chondroitin sulfate [21]. Its role in tumorigenesis and metastasis is thought to be through signaling pathways that regulate cell adhesion, migration, proliferation, differentiation and survival [22,23]. Histopathological studies of human gastric carcinoma have associated high CD44 expression with tumor invasion, lymph node metastasis and patient survival [24,25,26,27], although the expression of CD44 in CGC has so far not been investigated.

p16 protein inhibits cyclinD-CDK4/6, and previous studies of human gastric carcinoma have shown that loss of p16 expression has been associated with increased measures of malignancy and poor clinical outcome [28]. One previous study showed loss of expression of p16 in seventeen cases of CGC [29].

14-3-3σ is the focus of much research in human medicine, including gastric carcinomas [30,31]. 14-3-3σ protein regulates the G1/S and G2/M cell cycle checkpoints through sequestration of CDK4, CDK2, and CDK1, and thus prevents mitosis and allows DNA repair. It may act as a tumor suppressor [32] or it may serve as an oncoprotein. Previously, in veterinary species, 14-3-3σ has been reported as an oncoprotein in canine mammary and urinary bladder carcinomas [33,34]. The association and implication of 14-3-3σ with CGC will be examined later in this study.

The aims of this study were to provide an update on the signalment data and histopathological classification of a large case series of CGC, and to further investigate the potential association of chronic inflammation and the presence of *Helicobacter* spp. with cancer. Furthermore, using a subset of cases, the expression patterns of four proteins (E-cadherin, p16, 14-3-3σ and CD44) were studied to determine their potential involvement in CGC development.

## 2. Materials and Methods

### 2.1. Case Selection

The surgical biopsy databases at VPG Histology, United Kingdom, and the SIDAVE-University of Lleida, Spain were searched for cases of CGC from 2009 to 2019. All cases included relevant medical records (signalment, clinical history, gross description, microscopic description and original diagnosis), and tissues were received at the Royal Veterinary College (RVC) as formalin-fixed paraffin-embedded wax blocks. Individually identifiable owner information was redacted by both supplying institutions. Cases with insufficient tissue or where the diagnosis was not certain on review were excluded.

A control group of canine gastric biopsy samples was established from cases provided by VPG and from the RVC pathology archive. Control group samples were defined based on sample quality, absence of gastric carcinoma, and no previous history of gastric carcinoma. Controls were animals presenting with typical gastrointestinal clinical signs including vomiting, diarrhea and weight loss; however, no tumor was present on histopathological examination.

### 2.2. Histopathological Evaluation

Histological sections, cut at 4 μm and stained with hematoxylin and eosin, were produced from the provided paraffin blocks and were evaluated microscopically by three veterinary pathologists (A.H., A.S.-B. and S.L.P.). Each case was confirmed as gastric carcinoma and further classified according to the WHO standard into five categories: tubular, papillary, mucinous, SRC, or undifferentiated carcinoma [35,36]. The most frequent histological pattern served as the main classification criterion for each case.

Mucosal inflammation present within histological sections was assessed using modified World Small Animal Veterinary Association standards [8,37,38]. For the purposes of this study, only those parameters consistent with chronic inflammation (intraepithelial lymphocytes, lamina propria lymphocytes and plasma cells, and gastric lympho-follicular hyperplasia) were scored as normal—0, mild—1, moderate—2, and marked—3, following modified WSAVA criteria [37]. A total chronic inflammation score (TCIS, ranging from 0 to 9) was recorded as the sum of the three parameters. Regions of surface ulceration were avoided when assessing chronic inflammatory parameters.

Sections were stained with Warthin–Starry for *Helicobacter* spp. identification. Quantification of *Helicobacter* spp. was performed based on the presence or absence of bacteria in the mucosa using the following system: 0—no helicobacter found in the sample; 1—low numbers of helicobacter found (<15 bacteria per field of highest helicobacter density); and 2—high numbers of helicobacter found (>15 bacteria per field).

### 2.3. Immunohistochemistry

Immunohistochemical labeling for E-cadherin, CD44, p16 and 14-3-3σ was performed on 4-μm-thick sections mounted on positively charged slides (SuperFrost Plus; Menzel Gläser, Braunschweig, Germany). Antigen retrieval, labeling, and counterstaining were performed on a Bond-Max Autostainer (Leica Biosystems, Newcastle-upon-Tyne, UK) using the Bond Polymer Refine detection system (Leica Biosystems). Primary antibodies and retrieval conditions were as follows: p16 (PA0016, 1:100,Novocastra, Newcastle Upon Tyne, UK); pH 6.0 buffer (ER1, Leica Biosystems) for 20 min, 14-3-3σ (SC-100638, 1:40, Santa Cruz Biotechnology, Heidelberg, Germany); pH 6.0 buffer (ER1) for 20 min, E-cadherin (NCH-38, 1:100, Agilent, Stockport, UK); pH 6.0 buffer (ER1) for 20 min, and CD44 (ab157107, 1:50 Abcam, Cambridge, UK); pH 9.0 buffer (ER2, Leica Biosystems) for 20 min. Internal positive tissue controls for E-cadherin and CD44 were available on each section.

### 2.4. Evaluation of E-Cadherin, CD44, p16 and 14-3-3σ Immunolabeling

Analysis of immunolabeled samples was performed by 3 veterinary pathologists (A.H., A.S.-B. and S.L.P.), discrepancies were discussed by use of a multi-headed microscope, and a consensus was reached. In all cases, areas of ulceration and/or necrosis were avoided for interpretation.

The distribution and intensity of E-cadherin and CD44 labeling were analyzed for each case using a similar semiquantitative scoring system. The percentages of tumor cells, neoplastic acini and intravascular tumor emboli that expressed CD44 and E-cadherin were assessed. Labeling for CD44 was scored as follows: 0, no labeling; 1, 1–9% positive tumor cells; 2, 10–49% cells; and 3, 50–99% cells. Labeling for E-cadherin was scored as follows: 0, no labeling; 1, 1–9% positive tumor cells; 2, 10–49% cells; 3, 50–79% cells; and 4, 80–100% cells. The intensity of E-cadherin and CD44 labeling (0—negative, 1—mild, 2—moderate, and 3—strong) and whether labeling was membranous and/or cytoplasmic was also recorded [39,40,41]. A total immunohistochemical score (TIS) [39] for E-cadherin (ranging from 0 to 12) and TIS CD44 (ranging from 0 to 9) was calculated as the product of the distribution and intensity scores. Normal gastric surface epithelium served as a positive internal control for E-cadherin and lymphoid follicles, satellite glial cells and macrophages for CD44.

For p16, neoplastic cells with cytoplasmic and/or nuclear antigen expression were interpreted as immunopositive. An immunohistochemical score was assigned based on the intensity of staining (0—none, 1—weak, 2—moderate, and 3—strong) and percentage of positive tumor cells (0, 0% cells; 1, <25% cells; 2, 25–50% cells; 3, 51–75% cells; 4, >75% cells) [42]. The product of the intensity and distribution scores gave a TIS p16 ranging from 0 to 12. As a positive control, normal skin was used where epithelial cells within the basal layer of the epidermis exhibited weak immunoreactivity.

14-3-3σ labeling was assessed using a previously published semiquantitative scoring system [43]. The scores for the percentage (1, ≤10% cells; 2, 11–50% cells; and 3, >50% cells) and staining intensity (0, negative; 1, weak; 2, moderate; and 3, intense) of positive cells were recorded and a TIS 14-3-3σ (ranging from 0 to 9) was calculated as the product of these two parameters for each of the studied cases. Normal canine urinary bladder, which showed cytoplasmic immunolabeling of the urothelium, was used as a positive control.

### 2.5. Statistical Analysis

Results were analyzed for any significant relationship between the following: signalment (i.e., age, sex and breed); biopsy type (endoscopic vs. full-thickness biopsies); and histopathological features (tumor classification, chronic inflammation score, helicobacter identification, quantification, and localization). Statistical comparisons were performed using GraphPad Prism version 8.0 for Windows (GraphPad Software, San Diego, CA, USA). Bar graphs were constructed to depict the mean and standard error of the parameter assessed for each group. To test for the significance of a relationship between two categorical variables, a Fisher’s test was used to analyze the significance of contingency tables such as endoscopic vs. full-thickness biopsies for finding helicobacter. An unpaired *t*-test was used to compare the mean inflammation scores of different groups. For continuous data, the Student’s *t*-test, analysis of variance (ANOVA), or Mann–Whitney U analysis test was used, depending on whether the data were normally distributed and whether two, or more than two groups, were compared. The significance level for all statistical tests was set at *p* < 0.05. The expression of p16, 14-3-3σ, E-cadherin and CD44 were compared with histological subtype and features of malignancy.

## 3. Results

### 3.1. Case Details and Signalment

One hundred and eighty-two (182) cases of CGC were identified from the archives of VPG Histology and the University of Lleida and submitted to the Department of Pathobiology and Population Sciences at the Royal Veterinary College. Following the initial histopathological review, 149 were suitable for inclusion in the study based on sample quality, presence of gastric carcinoma, and amount of tumor present in the sample. Cases included 85 (57.0%) endoscopically obtained (mucosa only) samples, and 64 (44.0%) full-thickness biopsies collected by surgical excision. Of the 149 dogs, 69 (46.3%) were female, 75 (50.3%) male and 5 (3.4%) of unreported sex. Neuter status was known in 118 cases, with 88 (74.6%) neutered. Twenty-nine non-tumor control cases were included, 24 (82.8%) endoscopic and 5 (3.4%) full-thickness biopsies. The control group comprised 15 males (51.7%), 13 females (44.8%), and one of unreported sex (3.4%). For the carcinoma group the mean and median ages were 9.1 and 9 years, respectively (range 1–16 years), and for the control group were 7.5 and 7, respectively.

The Staffordshire bull terrier (22/149, 14.8%) was the breed most frequently affected by gastric carcinoma. Of the other non-cross breeds, Labrador retrievers (12, 8.1%), golden retrievers (10, 6.7%), Boxers (9, 6.0%), Border collies (8, 5.4%), rough collies (6, 4.0%), and Belgian shepherd dogs (6, 4.0%) were also commonly affected (Table S1). A similar range of breeds was seen in the control population, with Staffordshire bull terriers, Labrador retrievers, Border collies and Belgian shepherd dogs all represented. No statistical correlation with breed, sex and age was found with regards to each CGC subtype.

### 3.2. Histopathological Subtypes

**Figure 1:**
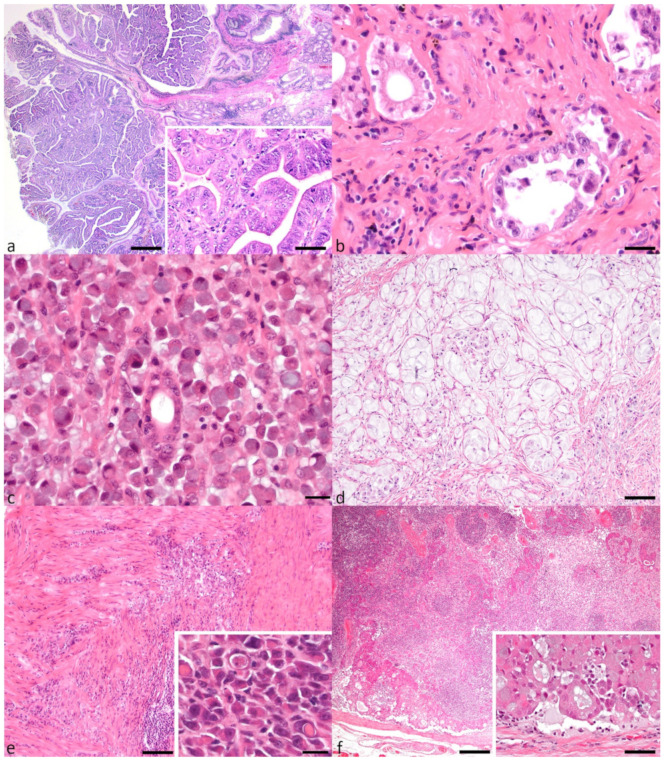
Gastric carcinoma subtypes and local lymph node metastasis. (**a**) Papillary adenocarcinoma with characteristic fibrovascular stalks supporting neoplastic cells that form fingerlike projections (bar = 500 μm). Inset shows increased mitoses (bar = 20 μm). (**b**) Tubular adenocarcinoma with neoplastic tubules lined by pleomorphic cells (bar = 20 μm). (**c**) Signet-ring cell carcinoma with characteristic signet-ring cells diffusely replacing gastric mucosa (bar = 20 μm). (**d**) Mucinous adenocarcinoma with abundant lakes of mucin and occasional small numbers of signet-ring cells (bar = 50 μm). (**e**) Undifferentiated adenocarcinoma with neoplastic cells replacing and dissecting through muscularis layers (bar = 200 μm). Inset showing entotic cell-in-cell (CIC) patterns in an undifferentiated adenocarcinoma (bar = 20 μm). (**f**) Lymph node metastasis of a tubular adenocarcinoma with multifocal intravascular neoplastic emboli (bar = 200 μm). Inset shows a high-power magnification of neoplastic tubule formation within the neoplastic emboli (bar = 20 μm).

Undifferentiated carcinoma was the most frequent carcinoma type overall, comprising 59/149 (39.6%) samples (Table 1). SRC carcinoma made up 47 (31.5%), tubular 32 (21.5%), and mucinous 10 (6.7%). The papillary type was rare, with only a single case (0.7%) (Figure 1) There were no significant associations identified between breed or sex and any of the five classifications of gastric carcinoma.

**Figure 2:**
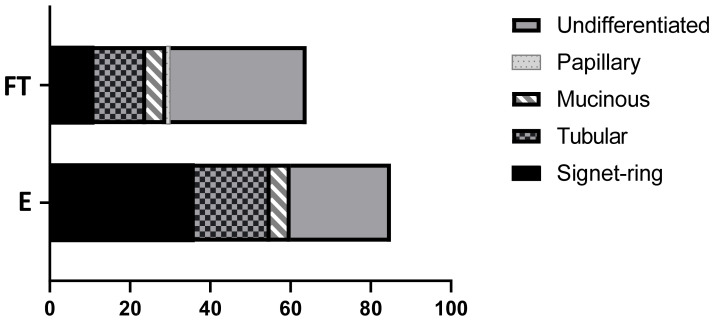
WHO classification of 149 canine gastric carcinomas separated by biopsy technique. Full-thickness surgical (FT), endoscopic (E).

Of the 85 endoscopic samples, 36 (42.4%) were SRC carcinoma, 25 (29.4%) were undifferentiated, 19 (22.3%) tubular, and 5 (5.8%) mucinous. Of the 64 full-thickness samples, 34 (53.1%) were undifferentiated carcinoma, 13 (20.3%) tubular, 11 (17.2%) SRC, and 5 (7.7%) mucinous. The single papillary adenocarcinoma was recorded amongst the full-thickness samples (1.5%) (Figure 2). The prevalence of SRC among endoscopic biopsies was significantly higher than among full-thickness biopsies (*p* = 0.0002). Comparisons between other subtypes in endoscopic and full-thickness biopsies did not show any statistical significance.

### 3.3. Inflammation and Presence of Helicobacter *spp.*

Mean total chronic inflammation score (TCIS) for the control group was 0.5/9 and for the carcinoma group was 2.8/9. Mean TCIS for the carcinoma group was significantly higher than that of the control group (*p* = 0.0001).

**Figure 3:**
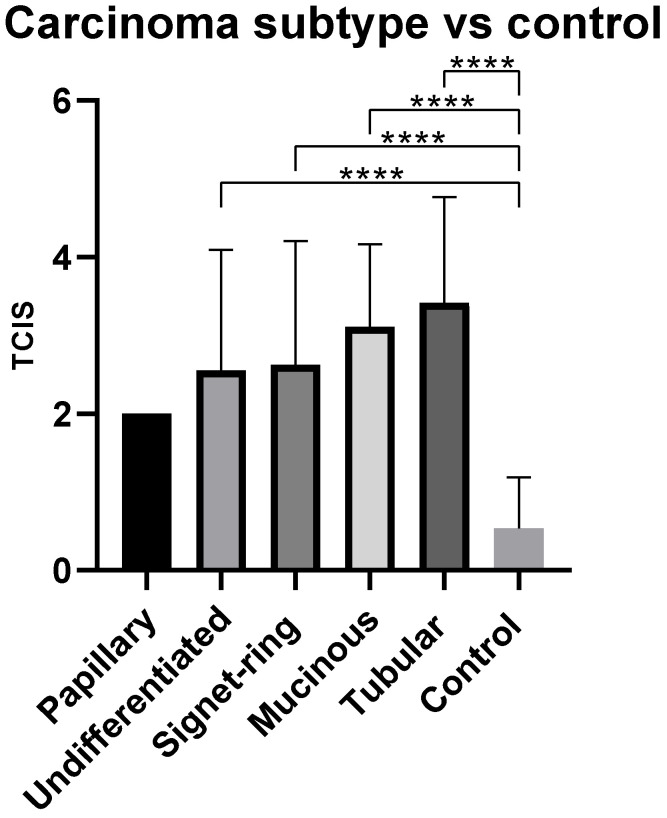
Mean total chronic inflammation score (TCIS) by carcinoma histological subtype. **** *p* < 0.0001.

Of all subtypes, tubular adenocarcinoma had the highest TCIS, 3.4, followed by mucinous—3.1, SRC—2.6, and undifferentiated carcinoma—2.5. Papillary adenocarcinoma had the lowest TCIS of 2.0. All subtypes (except for papillary) had a consistently significantly greater TCIS when compared with the control group (*p* = 0.0001) (Figure 3).

**Figure 4:**
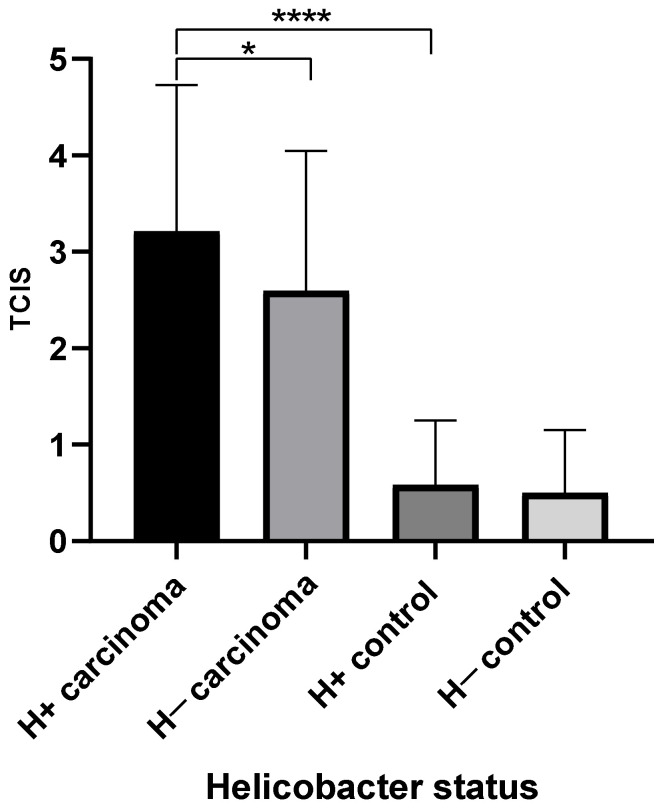
Mean total chronic inflammation score (TCIS) by helicobacter status for carcinoma and control groups. H+, helicobacter positive samples, H−, helicobacter negative samples.

Fifty out of 149 (33.5%) gastric carcinoma samples had observable *Helicobacter* spp., of which 19 (38%) had large numbers present. The TCIS of *Helicobacter* spp. positive samples was 3.2, versus 2.6 for *Helicobacter* spp. negative samples. These two means were found to be significantly different (*p* = 0.039) (Figure 4).

Thirteen out of 29 (44.8%) of the control samples had observable *Helicobacter* spp., but only 2 (15.4%) had large numbers present. The TCIS of *Helicobacter* spp. positive samples was 0.58, and helicobacter negative samples was 0.50. These two means were not significantly different.

### 3.4. Immunohistochemical Assessment of E-Cadherin, CD44, 14-3-3σ and p16

Twenty-two cases of gastric carcinoma, randomly selected from each histopathological subtype, were available for immunohistochemical assessment as follows: SRC (8, 36.4%), undifferentiated (7, 31.8%), tubular (5, 22.7%), papillary (1, 4.5%), and mucinous (1, 4.5%).

### 3.5. E-Cadherin Expression

**Figure 5:**
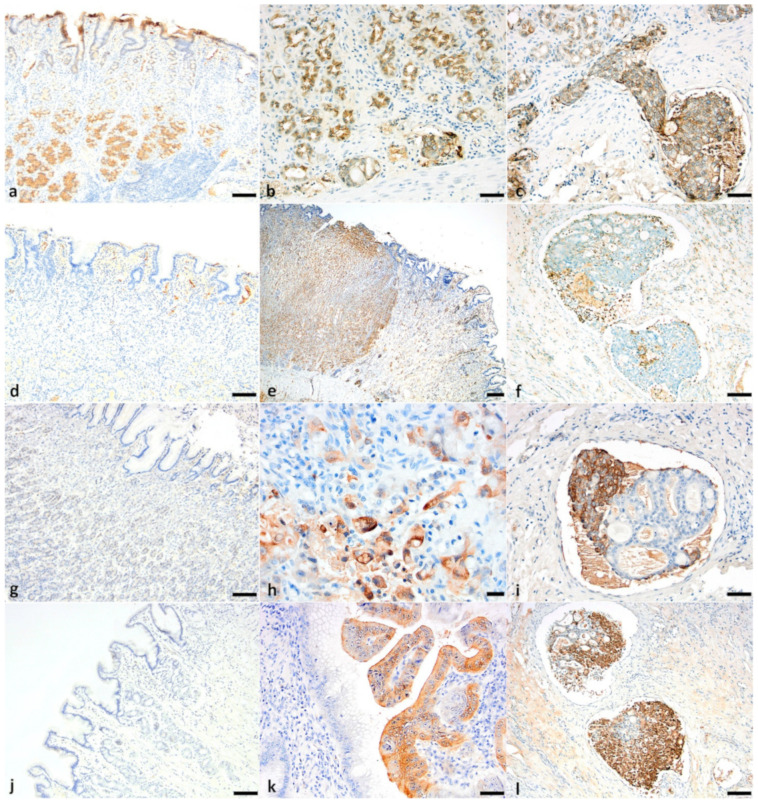
Canine gastric carcinoma immunohistochemistry for E-cadherin, CD44, 14-3-3σ, p16. (**a**) Expression of E-cadherin in normal gastric surface and glandular epithelium (bar = 100 μm). (**b**) Reduced E-cadherin expression (progressively cytoplasmic from membranous) in neoplastic tubules in a tubular adenocarcinoma (bar = 50 μm). (**c**) Poorly differentiated neoplastic cells in emboli showing cytoplasmic and membranous labeling for E-cadherin (bar = 50 μm). (**d**) Expression of CD44 in lymphocytes, macrophages and dendritic cells of normal gastric mucosa. (bar = 100 μm) (**e**) Strong membranous labeling of CD44 in sheets and chains of poorly differentiated neoplastic cells in an undifferentiated gastric carcinoma (bar = 200 μm). (**f**) Loss of membranous and cytoplasmic labeling of CD44 in tubules and strong membranous and cytoplasmic labeling in poorly differentiated cells of a neoplastic embolus (bar = 100 μm). (**g**) Normal epithelium showing no expression of 14-3-3σ (bar = 100 μm). (**h**) Strong cytoplasmic with occasional nuclear labeling of 14-3-3σ in nests of poorly differentiated neoplastic cells in an undifferentiated gastric carcinoma (bar = 20 μm). (**i**) Loss of cytoplasmic labeling of 14-3-3σ in tubules and cytoplasmic labeling in poorly differentiated cells of a neoplastic embolus (bar = 50 μm). (**j**) Normal epithelium showing no expression of p16 (bar = 100 μm). (**k**) Strong cytoplasmic labeling of p16 in neoplastic epithelium (bar = 50 μm). (**l**) Loss of cytoplasmic labeling for p16 in tubules and strong nuclear and cytoplasmic labeling in poorly differentiated cells of a neoplastic embolus (bar = 50 μm).

In normal gastric surface and glandular epithelium, E-cadherin labeling was strong (3/3 intensity score) and membranous (Figure 5a). Seventeen (of 22) tumors demonstrated positive immunolabeling for E-cadherin, with both membranous and cytoplasmic (17/22, 77.3%) and nuclear (2/22, 9.1%) labeling, and with an intensity greatest in intravascular emboli, where present. Five tumors did not show immunopositivity for E-cadherin (2 undifferentiated, 1 SRC, 1 mucinous and 1 tubular carcinoma). Where dysplastic epithelium was present, E-cadherin expression decreased in intensity and distribution (compared with normal), and labeling became progressively cytoplasmic (from membranous) (Figure 5b). The TIS E-cadherin for each tumor subtype was as follows: papillary—6.0; undifferentiated—4.6; SRC—4.3; tubular—3.2; and mucinous—0 (Table 2). Aberrantly increased expression and TIS of E-cadherin in intralymphatic/intravascular emboli, compared with both normal epithelium and immunopositive neoplastic cells, was observed in seven tumors (7/17; 3/7 undifferentiated, 1/8 signet-ring cell and 3/5 tubular carcinoma). E-cadherin was not expressed by neoplastic emboli composed of well-differentiated acinar structures (Figure 5c).

### 3.6. CD44 Expression

In the normal canine stomach, CD44 was expressed on the membrane of lymphocytes, macrophages, dendritic cells, and satellite glial cells (Figure 5d). Gastric epithelium, stromal tissue, smooth muscle fibers and matrix fibroblasts were negative. Positive immunolabeling (neo-expression) occurred in all gastric carcinoma cases. CD44 expression was cytoplasmic and membranous (5/22, 22.7%) or only membranous (9/22, 40.9%), with an intensity greatest in mucinous tumors (mean intensity score 2.5). Enhanced membranous expression was observed in those neoplastic epithelial cells that showed the greatest features of malignancy (pleomorphism and invasion), with a higher mean score in papillary, undifferentiated and tubular carcinomas (6, 4.7 and 4, respectively) (Figure 5e). Intravascular neoplastic emboli, present in 4/7 undifferentiated, 4/5 tubular and 3/7 SRC carcinomas, showed increased TIS compared to immunopositive neoplastic cells. Neoplastic cells in emboli exhibited membranous, or both cytoplasmic and membranous expression, in poorly differentiated neoplastic cells. Interestingly, there were neoplastic emboli that had solid nests and well-differentiated acinar arrangements, and, in these emboli, only the solid nests were strongly positive, while the well-differentiated embolic acini were negative (Figure 5f).

### 3.7. 14-3-3σ Expression

In a histologically normal canine stomach, 14-3-3σ was not expressed in any cell types, including epithelium, stromal tissue or lymphocytes (Figure 5g). Positive immunolabeling (neo-expression) occurred in 10 of 22 (45.4%) gastric carcinomas, 7/7 undifferentiated, and 3/5 tubular. TIS 14-3-3σ for undifferentiated and tubular carcinomas was 3.4 and 2.4, respectively. SRC, papillary and mucinous carcinomas were all negative for 14-3-3σ. Cytoplasmic and/or nuclear neo-expression of 14-3-3σ was present, with an intensity greatest in undifferentiated tumors (intensity mean 2.1, and total score 3.4). One case of undifferentiated carcinoma showed both nuclear and cytoplasmic labeling in neoplastic cells (Figure 5h). Where neoplastic emboli were present, TIS was higher compared to immunopositive neoplastic cells, and 14-3-3σ labeling intensity was increased in poorly differentiated and pleomorphic cells. However, when neoplastic cells formed intravascular acinar structures, 14-3-3σ was not expressed (Figure 5i).

### 3.8. p16 Expression

In a histologically normal canine stomach, p16 was not expressed (Figure 5j), but positive cytoplasmic and/or nuclear labeling was noted in regions of dysplastic epithelium (Figure 5k). p16 expression occurred in 19/22 (86.4%) carcinomas; 6/7 undifferentiated, 5/5 tubular, 1/1 papillary, 6/8 SRC, and 1/1 mucinous. Positive immunolabeling of pleomorphic cells and dysplastic tubules was found in all tumors, with high-grade expression in mucinous tumors (signet-ring cells in SRC and mucinous carcinomas were strongly immunopositive for p16). p16 exhibited cytoplasmic (11/22, 50%) and both nuclear/cytoplasmic (8/22, 36.36%) expression, with intensity greatest in mucinous tumors (intensity mean 3 and total score 9).

Where neoplastic emboli were present, TIS was higher compared to immunopositive neoplastic cells. Within intravascular and intralymphatic emboli, p16 intensity and cytoplasmic and/or nuclear expression was increased in poorly differentiated and pleomorphic cells. However, when neoplastic cells formed intravascular acinar structures, p16 was not expressed (Figure 5l). Similar findings were noted in the lymph node metastasis of one sample. The mean TIS p16 for undifferentiated, tubular, papillary, SRC and mucinous carcinomas was 4.3, 6.8, 6, 6.9 and 9, respectively.

## 4. Discussion

In this canine gastric carcinoma (CGC) study, using the WHO scheme, we have demonstrated that the most frequent subtype is the undifferentiated carcinoma, and the most common breed affected is the Staffordshire bull terrier. There is no sex predisposition, and the mean age is nine years. *Helicobacter* spp. presence was associated with increased chronic inflammation parameters and a greater chronic inflammatory score. We found that all markers showed variable expression for each subtype. CD44 and 14-3-3σ have not been previously investigated in CGC. 14-3-3σ was positive in undifferentiated, tubular and papillary carcinomas, and p16 expression was increased in mucinous and SRC carcinomas. E-cadherin and CD44 were variably expressed in all subtypes and were associated with criteria of malignancy. Within neoplastic emboli, acinar structures lacked expression of all markers, suggesting an independent molecular pathway that requires further investigation.

Of the 149 dogs included in this study from the UK and Spain, the mean and median age of 9.1 and 9 years, respectively, at the time of CGC diagnosis is in broad agreement with previously reported studies [3,6,7]. In this study, CGC affected male and female dogs roughly equally, with a slight preponderance towards males, as is consistent with previous reports [3]. The most commonly affected breed was the Staffordshire bull terrier, likely reflecting both the general population in the United Kingdom (only 2/22 Staffordshire bull terriers were from Spain) and previous reports of breed predisposition [7]. Breed predisposition to CGC is also reported in less common breeds, including Belgian shepherd dogs and rough collies, which also appeared with an increased frequency in this study [3,6].

Using the WHO classification for domestic animals, cases were divided into five categories based on the predominant histological features and the principal cell type of the tumor. All histological subtypes of carcinoma (SRC, tubular, mucinous, papillary and undifferentiated) were recorded, although squamous cell carcinoma, another CGC subtype, was not present in this study [44]. Undifferentiated carcinoma was the most frequent subtype, followed by SRC, contrary to the previous studies reporting an increased frequency for the tubular subtype, similar to humans [45,46]. This could likely reflect a geographic variation in gastric adenocarcinoma incidence.

Signet-ring cell carcinomas were the most frequently diagnosed type by endoscopy, whereas undifferentiated carcinomas were the most frequently diagnosed in full-thickness samples. The most likely reason behind this difference is that, histologically, the diagnosis of SRC subtype is based upon identification of the characteristic isolated or small groups of malignant cells containing intracytoplasmic mucin with an eccentric nucleus (signet-ring cells) within the mucosa, and hence, diagnosis from an endoscopically retrieved (mucosa only) sample is possible. The diagnosis of other histopathological subtypes typically requires the evaluation of invasion beyond the mucosa, and thus, given the relatively high number of full-thickness biopsies in this study, this may have influenced the predominant subtype. Additionally, signet-ring cells were also present in other subtypes, i.e., mucinous and undifferentiated carcinoma; however, the predominant histological features and pattern did not favor a diagnosis of SRC carcinoma. This suggests that endoscopic samples alone can lead to diagnostic pitfalls between different subtypes [47,48,49,50]. In humans, novel techniques such as endoscopic surgical dissection are recommended in subtypes like SRC that spread subepithelially in the margins [51]. In combination with future novel imaging techniques, these findings should be taken into consideration to determine the appropriate sampling and therapeutic approach [52].

Cancer-related inflammation is one of the hallmarks of neoplasia, and once cancer develops, there is evidence of a substantial shift in the microenvironment affecting the immune response [53,54]. A simplified histopathologic scoring system was adapted to reduce variability in the diagnostic interpretation [37]. This scoring system captured and quantified chronic inflammatory changes in the gastric mucosa [55]. The TCIS for the carcinoma group, and individually for each CGC subtype (independent of helicobacter presence), was significantly greater when compared to the control group’s TCIS. Thus, there is a clear correlation between chronic inflammation and carcinoma for most CGC subtypes.

Given the link between the presence of helicobacter, inflammation and neoplasia in humans we aimed to quantitatively assess helicobacter presence in CGCs. In previous studies *Helicobacter* spp. have been reported in both neoplastic and non-neoplastic canine stomachs [56,57]. In this study, the TCIS of the carcinoma group with a concomitant presence of helicobacter was significantly greater compared to the helicobacter-negative carcinoma group. Furthermore, larger numbers of helicobacter were found in carcinoma cases than in control cases. Although the helicobacter presence does not prove a definite role in CGC pathogenesis, there is a clear association between increased chronic inflammation and higher numbers of helicobacter in the tumor cases versus controls. Whether the presence of *Helicobacter* spp. is associated with tumor development is not exactly clear, and thus further studies, perhaps to examine particular species of helicobacter in association with CGC, would be needed.

To understand the molecular pathways and investigate the cellular origin of CGC, the expression of cellular adhesion molecules (E-cadherin and CD44) and cell cycle regulators (p16 and 14-3-3σ) were investigated in a representative proportion of cases. CD44 and 14-3-3σ have not previously been studied in CGC.

E-cadherin controls cell motility and suppresses tumor growth and metastasis [58]. Immunohistochemical examination of E-cadherin in all tumor subtypes identified abnormalities of expression and localization. Dysplastic surface and glandular epithelia in immunopositive cases revealed a reduction in E-cadherin expression. Pleomorphic cells in the undifferentiated subtype showed E-cadherin expression, albeit in decreased intensity compared to the normal epithelium. Subcellular localization of E-cadherin was observed in the majority of neoplastic cells. Intracytoplasmic sequestration and the accumulation of E-cadherin have been previously associated with abnormalities in the intracytoplasmic transport and reuptake mechanisms of the molecule [59,60]. Aberrant nuclear expression of E-cadherin in humans has been previously described in several tumor types including gastric and colorectal carcinomas [19,61]. Similarly, increased intensity intracytoplasmic and nuclear expression was noted in the neoplastic emboli composed of pleomorphic neoplastic cells forming solid nests. Surprisingly, intravascular acinar structures did not express E-cadherin. Furthermore, tubular adenocarcinoma, which is composed of acini and tubules, had the lowest TIS compared to other subtypes. This could represent an inverse association between the reduction or loss of E-cadherin with an increasing degree of differentiation, where well-differentiated structures switch off and pleomorphic cells switch on or increase the expression of E-cadherin. Previous studies in human gastric adenocarcinoma showed that abnormalities of E-cadherin localization (internalization) in neoplastic cells lead to decreased adhesion, thus favoring invasion [59,60]. Neoplastic cells lacked E-cadherin expression in discohesive subtypes like mucinous adenocarcinoma, which is in accordance with a recent study [20].

In the context of these findings, we postulate that the reduction or loss of E-cadherin expression on the cell membrane could potentially facilitate invasion, and that the re-expression of E-cadherin, including on the cell membrane, in neoplastic emboli could lead to the development of solid cohesive intravascular structures, enhancing their survival. These findings reinforce the notion that E-cadherin in CGC is a context-dependent adhesion molecule that can either be up- or down-regulated or re-expressed, depending on the stage of tumor progression [62]. Further studies analyzing ligands of E-cadherin and possible mutations are required to clarify its precise role in CGC.

CD44 serves as a signaling platform, with transmembrane and cytoplasmic domains as a co-receptor for various types of cell surface receptors that modulate cell adhesion, migration, proliferation, differentiation and survival [63,64,65,66]. In humans, its role in the adaptive plasticity and survival of cancer cells in processes like epithelial to mesenchymal transition, invasion and metastasis has been widely studied [67,68]. In CGC, CD44 was variably expressed in all tumor subtypes. Similar to E-cadherin, the expression of CD44 was enhanced in pleomorphic neoplastic cells and was absent in well-differentiated acinar structures within neoplastic emboli. In the context of these findings, we postulate that CD44 expression could potentially facilitate invasion and expression in neoplastic emboli and, alongside E-cadherin, could lead to the development of cohesive intravascular structures, enhancing their survival. In previous studies in humans, CD44 positive cells in gastric cell lines were associated with increased chemoresistance and invasiveness [66]. Additionally, CD44 positive gastric tumors were associated with larger tumor size, a lower grade of differentiation, tumor relapse, lymph node invasion, distant metastasis and reduced survival [63,64,65,66]. It seems that CD44 may represent an important biomarker and a promising therapeutic target in canine gastric carcinomas.

14-3-3σ is a cell cycle regulator that may serve as either a tumor suppressor or an oncogene involved in tissue invasion and metastasis. Histological features of malignancy have been previously associated with the overexpression and/or neo-expression of the protein [69,70]. The role of this protein as a tissue differentiation marker and as an oncoprotein in veterinary medicine has been previously studied in canine mammary, urinary bladder, renal cell and equine penile squamous cell carcinomas [33,34,71,72]. In the current study, immunohistochemical analysis revealed the absence of 14-3-3σ immunolabeling in a normal stomach and in SRC and mucinous carcinomas. The latter finding was consistent for all cases of the two subtypes and likely demonstrates a 14-3-3σ independent molecular pathway in carcinogenesis. In contrast, tubular and undifferentiated subtypes showed strong intracytoplasmic neoexpression, and occasionally nuclear expression of 14-3-3σ. The single papillary carcinoma had weak intracytoplasmic neoexpression. Undifferentiated carcinomas demonstrated strong intracytoplasmic, and occasionally nuclear, expression in the most pleomorphic neoplastic cells. Similarly, in those tumors with neoplastic emboli formed by pleomorphic cells forming solid nests, there was strong intracytoplasmic and occasionally nuclear expression of 14-3-3σ. However, acinar structures within the intravascular emboli lacked expression of 14-3-3σ. Aberrant nuclear expression of 14-3-3σ, in a subset of cases of renal cell carcinomas, was associated with a malignant phenotype and shorter survival rate [43]. Thus, we hypothesize, in view of these findings, that expression of 14-3-3σ is associated with features of malignancy, and that neoexpression of the molecule is essential in the stage of intravascular invasion. Expression of the protein in the extracellular milieu, as previously demonstrated for other canine carcinomas, was not observed in this study [72].

The protein p16 acts as a tumor suppressor and a cell cycle regulator, by slowing progression from the G1 to S phase through the inhibition of cyclinD-CDK4/6. Its role and possible relationship to tumor progression and prognosis have been studied in a variety of human tumors [73,74,75]. In human gastric carcinomas, loss of p16 expression has been associated with malignant characteristics and poor prognosis [28,76,77]. In the CGCs studied here, the expression of p16 seemed to be an event common to all subtypes, and thus appears fundamental for neoplasia development, with expression most strong in mucinous and SRC carcinomas. Half of the tumors showed cytoplasmic, and 36.7% both nuclear and cytoplasmic, immunolabeling. Within neoplastic emboli, expression was cytoplasmic and only in one tubular carcinoma case was both cytoplasmic and nuclear. Similar to the other markers, when acinar structures were present within the emboli, p16 expression was absent. These results contradict a previous canine study, where lack of p16 expression in tubular, SRC and undifferenced subtypes was significantly associated with histological criteria of malignancy in 14 cases [29]. In humans, the overexpression of p16 is associated with mutations in genes encoding retinoblastoma protein (Rb) and p53, and is considered to be a mechanism to arrest the uncontrolled proliferation caused by failure of the Rb pathway [78]. Furthermore, the significance of the p16 expression within the cell is not clear, and few studies have associated cytoplasmic expression with malignant features [79]. Currently, similar to humans, the significance of p16 expression patterns in CGC remains unclear [78].

## 5. Conclusions

In conclusion, CGC affects female and male dogs in relatively equal proportions, with a mean age of nine years, and the highest frequency in Staffordshire bull terriers. The most common CGC histological subtype was the undifferentiated carcinoma. CGCs were associated with increased chronic inflammation parameters and with a greater chronic inflammatory score when *Helicobacter* spp. were present. Understanding the altered molecular pathways associated with gastric carcinoma development including its histological subtype remains a challenge. The significance of the findings from the cell cycle regulators and cell adhesion molecules in the subtypes examined, should be combined with clinicopathological data and additional molecular analysis to further understand the molecular mechanisms and similarities between dogs and humans.

## Figures and Tables

**Table 1:** Canine gastric carcinoma subtype by sex.

Gastric Carcinoma Subtype	Male	Female	Not Recorded	Total
Undifferentiated	32	26	1	59 (39.6%)
Signet-ring	22	24	1	47 (31.5%)
Tubular	17	12	3	32(21.5%)
Mucinous	4	6	-	10 (6.7%)
Papillary	0	1	-	1 (0.7%)
Total	75	69	5	149

**Table 2:** Mean total immunohistochemical score by gastric carcinoma subtype. The same score for neoplastic vascular emboli, where present, is given in parentheses.

Protein	Canine Gastric Carcinoma Subtype				
	Undifferentiated	Signet-Ring	Mucinous	Tubular	Papillary
E-cadherin	4.6 (9)	4.3 (6)	0	3.2 (10)	6
CD44	4.7 (6.3)	3 (6.3)	4	2.4 (3.6)	6
14-3-3σ	3.4 (9)	0	0	2.4 (9)	0
p16	4.3 (9)	6.9	9	6.8 (10.5)	6

## Data Availability

The data presented in this study is contained within the manuscript and Supplementary Table S1.

## Supplementary Materials

The following are available online at https://www.mdpi.com/article/10.3390/ani11051409/s1, Table S1: 149 cases of canine gastric carcinoma by sex and breed frequency.
