# Supplementary material for: Canine Gastric Carcinomas: A Histopathological and Immunohistochemical Study and Similarities with the Human Counterpart
Source: Animals (Basel). 2021 May 14;11(5):1409. doi: 10.3390/ani11051409 (PMC8156491; doi:10.3390/ani11051409)
Supplement: Supplementary file 1 [file animals-11-01409-s001.zip › animals-1156810-supplementary.pdf]

animals

MDPI

Supplementary table 1: 149 cases of canine gastric carcinoma by sex and breed frequency.

Breed	M	F	Total	Breed%
1. Staffordshire Bull Terrier	16	6	22	14.8
2. Cross breed	9	6	15	10.1
3. Labrador Retriever	3	8	12	8.1
4. Golden Retriever	4	6	10	6.7
5. Boxer	3	5	9	6
6. Border Collie	1	6	8	5.4
7. Belgian Shepherd Dog	1	4	6	4
8. Rough Collie	3	3	6	4
9. West Highland White Terrier	3	2	5	3.4
10. Collie	X	3	3	2
11. Dalmatian	2	1	3	2
12. Leonberger	1	2	3	2
13. Bearded Collie	1	1	2	1.3
14. Husky	2	X	2	1.3
15. Jack Russell Terrier	2	X	2	1.3
16. Labradoodle	1	1	2	1.3
17. Lhasa Apso	1	1	2	1.3
18. Standard Poodle	1	1	2	1.3
19. Cocker Spaniel	2	X	2	1.3
20. Chow Chow	2	X	2	1.3
21. German Shepherd Dog	1	1	2	1.3
22. English Springer Spaniel	1	X	1	0.7
23. Basset Hound	1	X	1	0.7
24. Blue Heeler	X	1	1	0.7
25. Bull mastiff	X	1	1	0.7
26. Cavalier King Charles Spaniel	1	X	1	0.7
27. Dachshund	1	X	1	0.7
28. English bull terrier	1	X	1	0.7
29. Flat-Coated Retriever	X	1	1	0.7
30. French Bulldog	1	X	1	0.7
31. Giant Schnauzer	X	1	1	0.7
32. Gordon Setter	X	1	1	0.7
33. Irish Red Setter	X	1	1	0.7
34. Keeshond	X	1	1	0.7
35. Lurcher	X	1	1	0.7
36. Miniature Schnauzer	X	1	1	0.7
37. Newfoundland	X	1	1	0.7
38. Norwegian Elkhound	1	X	1	0.7
39. Parson Russell Terrier	Not recorded	Not recorded	1	0.7

40.	Pointer	1	X	1	0.7
41.	Rhodesian Ridgeback	1	X	1	0.7
42.	Samoyed	1	X	1	0.7
43.	Scottish Terrier	1	X	1	0.7
44.	St. Bernard	1	X	1	0.7
45.	Tibetan Spaniel	X	1	1	0.7
46.	Weimaraner	1	X	1	0.7
47.	Spitz	1	X	1	0.7
48.	American Bulldog	1	X	1	0.7
49.	Epagneul breton	X	1	1	0.7
50.	Shar-pei	1	X	1	0.7
	**Total**	-	-	**149**	**100%**
